# Mood Induction in Children: Effect of the Affective Valence of a Text on Phonological Working Memory

**DOI:** 10.5709/acp-0162-z

**Published:** 2014-09-30

**Authors:** Michaël Fartoukh, Lucile Chanquoy, Annie Piolat

**Affiliations:** 1 University of Nice Sophia Antipolis, CNRS, BCL, UMR 7320, 06300 Nice, France; 2 Center for Research in the Psychology of Cognition, Language & Emotion, EA, 3273, Aix-Marseille University, France

**Keywords:** children, text valence, emotion, mood, phonological working memory

## Abstract

The influence of mood on working memory capacity has been investigated in adults, albeit with conflicting results, but remains relatively unexplored in children. The present study examined the effect of a mood induction procedure on phonological working memory capacity in fourth and fifth graders. An initial working memory span test was followed first by a collective mood induction procedure, then by a second working memory span test. Results showed an effect of mood induction procedure on phonological working memory performances, with decreasing scores in the case of negative mood. These results suggest that, in certain contexts and situations, negative emotion has an impact on children’s cognitive abilities.

## Introduction

During the course of everyday life events, we experience many emotions that may temporarily, or even permanently, influence our mood or emotional state. Conversely, our emotional state can sometimes influence how we process information, reason, or exchange and interact with others. Thus, despite their adaptive role, emotions may impair our ability to monitor and understand the information we have to process (Blanchette & Richards, 2010). More specifically, emotions may affect both cognitive abilities and working memory (WM) capacity (Kensinger & Corkin, 2003; Martin & Kerns, 2011; Spies, Hesse, & Hummitzsch, 1996). Depending on the context and the nature of the task being performed, positive and/or negative emotions may have a facilitating effect or indeed no effect at all on WM performances (Levens & Phelps, 2008). Even so, many studies have revealed an inhibitory or disruptive effect of emotions on WM storage and retrieval (Gotoh, 2008; Oaksford, Morris, Grainger, & Williams, 1996; Phillips, Smith, & Gilhooly, 2002; Spies et al., 1996).

Very little is yet known about the mechanisms responsible for the effects of emotion on cognition. One possibility, put forward by Martin and Kerns (2011), is that positive mood, by virtue of its euphoriant nature, increases the spread of activation of items in WM. This activation makes it more difficult for a system with limited resources to maintain and focus attention on particular items (Davelaar, Goshen-Gottstein, Ashkenazi, Haarmann, & Usher, 2005). Thus, the activation that occurs in the wake of a positive mood lowers performances on WM span tests. For their part, Martin and Kerns (2011) found that a positive mood could decrease the amount of WM storage available for a running span task. Similarly, using the Tower of London task, Oaksford et al. (1996) showed that a positive mood has negative effects on WM resources and capacity.

Concerning negative mood, although its effects appear to be substantially similar to those of positive mood, results diverge when it comes to identifying the causes, the most likely suspect here being intrusive thoughts (Ellis & Ashbrook, 1988; Ellis & Moore, 1999; Eysenck, Derakshan, Santos, & Calvo, 2007). Studies have shown that forming intrusive thoughts (i.e., thoughts not relevant to the cognitive activity in progress) takes up a large proportion of the resources available in WM (Teasdale et al., 1995). The activation aroused by negative mood therefore increases cognitive load by occupying space in WM and focusing attentional resources on the emotional state being experienced.

According to Phillips et al. (2002), a mood induction procedure may have different effects on WM, depending on the individual’s WM capacity. Participants with below average WM capacity have greater difficulty managing the additional cognitive load engendered by a positive or a negative mood in a problem-solving situation (e.g., Tower of London). All these elements lead us to infer that, in some cases, emotions can be likened to a dual task, in terms of demand on memory resources, as postulated by several authors (Ellis & Ashbrook, 1988; Ellis & Moore, 1999; Spies et al., 1996). In addition, the effect of a mood induction procedure may differ according to the participants’ WM capacity (Phillips et al., 2002). This effect may be greater when individuals have relatively limited processing abilities that are still developing, as is the case of school-age children.

### Children’s Working Memory

WM is described in the literature as a flexible system constrained by limited resources that can be allocated to either the storage or the processing of information. For some authors, WM is the active component of long-term memory (Cowan, 1995). However, according to Baddeley (2000), WM consists of four main components: three slave systems (phonological loop, visuospatial sketchpad and episodic buffer) managed by a central executive. The latter forms the heart of the system, allowing for the coordination of different kinds of information, the inhibition of information that is irrelevant to the task, and the modification or adaptation of ongoing strategies. In addition, it oversees, regulates and controls complex cognitive processes, with the help of the three slave systems. The limited-capacity subsystems (phonological loop and visuospatial sketchpad) are used for the temporary retention of information.

WM development in children has been extensively studied (for a review, see Hitch, 2006). According to Gathercole, Pickering, Ambridge, and Wearing (2004), WM capacity undergoes steady development between the ages of 4 and 15 years. The increase in performances is almost linear, and all the WM components seem to be in place by around the age of 6 years. The capacity of each component changes over time and the relationship between them gradually strengthens. The similarity in WM structure between adults and children raises questions about the possible effects of mood induction on WM capacity in children.

### Effect of Emotion on Cognition in Children

Although little research has been devoted to studying the effect of emotional induction on children’s cognitive performance, some clues suggest that this effect could be similar or close to that observed in adults. For example, Masters, Barden, and Ford (1979) showed that the induction of an emotional state affects the learning abilities of 4-year-olds. Positive mood had a positive effect, improving abilities, while negative mood had the opposite effect. Similar results were also reported by Rader and Hughes (2005) for positive mood, in the context of a visual problem performed by 6-7 year-olds. Moreover, Greene and Noice (1988) showed that positive emotional induction resulted in greater cognitive flexibility among eighth graders. Their responses to Duncker’s candle problem were better than those of the children in the control condition.

Taken together, these studies lead us to think that mood induction-be it positive or negative-has an effect in children. As seems to be the case with adults, emotion may sometimes bring about cognitive overload in certain contexts. However, to our knowledge, no research has yet examined a possible effect of emotion on phonological WM in children.

### The Present Experiment

Given that positive or negative mood can undermine or interfere with WM capacity (Ellis & Ashbrook, 1988; Kensinger & Corkin, 2003; Martin & Kerns, 2011; Spies et al., 1996), we explored for the first time the effect of a mood induction procedure on phonological WM capacity in children. We chose to work with fourth and fifth graders, the last grades of French primary school, because of their relatively advanced emotional maturity and their comprehension capacity. On the basis of our first hypothesis that the oral reading of texts with a positive or negative emotional valence should affect children’s phonological WM, as assessed by a WM test, we predicted that children under positive or negative mood induction would perform more poorly on the WM test than children under neutral mood induction. Mood induction can be achieved by reading stories with contrasting emotional content. This is one of the most efficient methods of inducing mood, in both adults (Westermann, Spies, Stahl, & Hesse, 1996) and children (Brenner, 2000; Rader & Hughes, 2005).

In addition, we predicted an interaction effect between mood induction and WM capacity (Phillips et al., 2002). We expected the additional cognitive load resulting from the positive or negative mood induction to have a greater impact on children with low WM capacity, regardless of children’s grades. Indeed previous studies already showed that fourth and fifth graders cannot be distinguished considering their WM capacities (e.g., Fartoukh, Chanquoy, & Piolat, 2012; Gathercole et al., 2004).

## Method

### Participants

The study was carried out with 54 fourth graders (31 girls, 23 boys, *M*_age_ = 10 years, age range: 9.5-11.4 years) and 58 fifth graders (32 girls, 26 boys, *M*_age_ = 11 years, age range: 10.1-12.2 years), without any problems in learning and receptive verbal ability, drawn from primary schools in Southeast France. All the children participated on a voluntary basis and the children’s parents gave their written informed consent. Children were randomly allocated to one of three mood induction conditions.

### Material

Mood (e.g., positive, negative, or neutral) was induced by reading the children three texts adapted to primary school children with contrasting emotional valences that had proved effective in inducing a congruent mood (Cuisinier, Sanguin-Bruckert, Bruckert, & Clavel, 2010; Fartoukh, Chanquoy, & Piolat, 2014). The positive text was about a famous funny boy named “Le petit Nicolas” which went on holidays with friends. They discovered the countryside, a farm and its animals. The negative text told the story of a lonely man lost in a snowy countryside. Unable to light a fire, the man fell down because of the cold weather. The neutral text was about a promenade in the mountain, and a farm nearby.

The phonological WM span task took the form of the Letter-Number Sequencing subtest of the French version of the Wechsler Intelligence Scale for Children (WISC-IV; Wechsler, 2005). This subtest comprises 10 sets of three trials, each featuring letters and numbers (2-8 items). At the end of each trial, participants have to recall first the numbers (in ascending order), then the letters (in alphabetic order). The score is determined by the number of correctly recalled trials (i.e., all the numbers and letters recalled in the correct order). For the purposes of the experiment, the material was modified to meet the constraint of a collective and written assessment. Only the first two trials in each of the 10 sets were kept, and these were used to form two sets of 10 trials each. To score one point, in a trial the children had to write down all the dictated numbers in ascending order, then all the dictated letters in alphabetic order, without making any mistakes. The maximum possible score in each set was 10 points.

### Procedure

Data were collected within classrooms by the same experimenter. After a short description of the activities (listening to a story and phonological WM span task), the first WM measure was taken. Children were instructed to place a screen between themselves and their neighbor (to avoid copying) and only write when the experimenter had finished reading out all the numbers and letters. Immediately after this test, a text (positive, negative, or neutral) was read out by the adult. A second WM measure was then taken. According to Brenner (2000), the effects of a mood induction procedure in children dissipate relatively quickly (5 min). As our mood induction procedure was attested in other studies (Cuisinier et al., 2010; Fartoukh et al., 2014), we therefore decided against administering an emotional state questionnaire, in order to avoid diluting the effects of the mood induction procedure by introducing an additional task. Finally, according to their performances in the first WM measure, children were divided in two WM capacity groups: low WM (scores between 4 and 6) and high WM (scores higher than 6).

## Results

Using the first WM measure as the dependent variable, a preliminary analysis of variance (ANOVA) was conducted to check firstly that all Mood Groups (positive, negative, and neutral) and both Grade Levels (fourth and fifth) had equivalent phonological WM capacity, secondly that WM Capacity Groups (low vs. high) had different phonological WM capacity. The 3 (Mood Group) × 2 (Grade Level) × 2 (WM Capacity) ANOVA for independent groups revealed no main effect of Mood Groups (*M*_positive_ = 6.41, *M*_negative_ = 6.42 and *M*_neutral_ = 6.60), *F*(2, 100) = 1.32, *MSE* = 0.23, *p* = .27, and no main effect of Grade Level (*M*_fourth_ = 6.35 and *M*_fifth_ = 6.60), *F*(1, 100) = 1.70, *MSE* = 0.23, *p* = .19. Conversely, the two WM groups had significantly different scores (Mlow = 5.73 vs. Mhigh = 7.25), *F*(1, 100) = 259.03, *MSE* = 0.23, *p* < .0001, ƞ_p_^2^ = .72.

Concerning the effect of the mood induction procedure on children’s phonological WM performances, we performed a 3 (Mood Group) × 2 (WM Capacity) × 2 (Time of Measurement: before vs. after induction) ANOVA with repeated measures on the last factor (results in Table 1).

**Table 1.** Mean Scores and Standard Deviations on the WM Span Test

		Time of Measurement	
Mood group	WM capacity	Before induction	After induction
Positive	Low	5,85 (0,35)	5,66 (0,85)
	High	7,20 (0,56)	6,86 (0,63)
Negative	Low	5,58 (0,61)	5,17 (1,07)
	High	7,22 (0,42)	6,77 (0,87)
Neutral	Low	5,72 (0,46)	5,83 (0,95)
	High	7,30 (0,47)	7,34 (0,71)

*Note*. Standard deviations in parentheses.

The results revealed a main effect of Mood Group (*M*_neutral_ = 6.55 vs. *M*_negative_ = 6.19 vs. *M*_positive_ = 6.39), *F*(2, 106) = 3.84, *MSE* = 0.63, *p* < .03, ƞ_p_^2^ = .06. The main effect of WM Capacity was significant, *F*(1, 106) = 188.87, *MSE* = 0.63, *p* < .0001, ƞ_p_^2^ = .64. The low WM capacity group performed lower than the high WM capacity group (5.64 vs. 7.11). The main effect of Time of Measurement was also significant, *F*(1, 106) = 6.57, *MSE* = 0.34, *p* < .02, ƞ_p_^2^ = .05. The scores fell between the two measures (*M*_WM1_= 6.48 vs. *M*_WM2_= 6.27).

**Figure 1.**
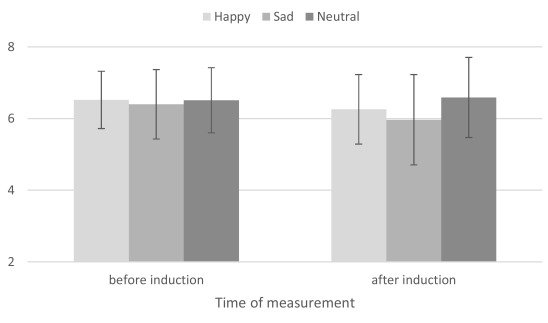
Interaction between Mood Group and Time of Measurement.

The interaction between Mood Induction and Time of Measurement, *F*(2, 106) = 3.62, *MSE* = 0.34, *p* < .04, ƞ_p_^2^ = .06, was significant (see Figure 1). More precisely, the results of the neutral Mood Group (*M*_WM1_= 6.51 and *M*_WM2_= 6.59), *F*(1, 106) = 0.34, *MSE* = 0.34, *p* = .85, and the positive Mood Group (*M*_WM1_= 6.52 and *M*_WM2_= 6.26), *F*(1, 106) = 3.44, *MSE* = 0.34, *p* = .07, did not change significantly between the two measures. By contrast, the results of the negative Mood Group were significantly lower after the mood induction (*M*_WM1_= 6.40 and *M*_WM2_= 5.97), *F*(1, 106) = 9.19, *MSE* = 0.34, *p* < .01, ƞ_p_^2^ = .21. The iteractions between Mood Induction and WM Capacity, *F*(2, 106) = 0.93, *MSE* = 0.63, *p* = .39, WM Capacity and Time of Measurement, *F*(1, 106) = 0.26, *MSE* = 0.34, *p* = .61, and between Mood Induction, WM Capacity and Time of Measurement, *F*(2, 106) = 0.04, *MSE* = 0.34, *p* = .96, were not significant.

## Discussion

In order to pinpoint the effect of emotions on phonological WM performances in school-age children, we administered an adapted WM span task before and after a mood (positive, negative, or neutral) induction procedure. Our first hypothesis predicted that reading texts of contrasting emotional valence (positive vs. negative) to the children would have an effect on their phonological WM performances. This hypothesis was partially validated. The patterns resulting from the induction of negative mood differed from those resulting from neutral and positive mood induction. More specifically, the neutral and positive mood group’s scores did not significantly change after the mood induction procedure. By contrast, there was a decrease in the negative mood group’s scores. This suggests that negative mood may impact the phonological WM resources available (Ellis & Ashbrook, 1988; Spies et al., 1996) but contrary to Ellis and Moore’s (1999) suggestions, more resources would appear to be consumed in the case of a negative mood than in the case of a positive mood. Another explanation could be found in the environment that characterized the study. This study was carried out in the classroom and had set children in a test situation. The stress of participating in such a study might have reduced the effect of inducing positive mood. In other words, the positive mood induced by the text might have been neutralized thus leading to no effect at all. Therefore, the positive induction procedure could not be as powerful as expected because of the environmental constraints of the testing situation. Consequently, no effect of the positive mood condition could be found.

Our second hypothesis predicted an interaction between mood induction and WM capacity. We expected the additional cognitive load resulting from the positive or negative mood induction to have a greater impact on children with low WM capacity (Phillips et al., 2002). Contrary to our expectations, the interaction was not significant. Children with both low and high WM capacities had comparable results, regardless of the mood induction.

This latter point led us to wonder about the limitations of our study. First, we wondered about the way the WM test was administered. Because we wanted to create a situation as close as possible to that in a classroom, we decided to use a collective procedure instead of an individual one that could have been better to control participants’ attention. A second limitation could be attributed to the way in which the mood was induced, namely, reading children three texts adapted to primary school with contrasting emotional valences. Of course, there are other mood induction procedures such as music or movies. The effect of these mood induction procedures on WM should be investigated in future studies. Finally, a third limitation concerned the fact that in order to avoid diluting the effects of the mood induction procedure, we decided not to measure the mood during the experiment. Future studies need to overcome such a drawback and make sure to check mood manipulation without influencing mood induction. A solution could be to interview children after the last WM task.

Despite these limitations, for the very first time, these results, in line with some others observed in adults, allowed us to demonstrate a link between mood induction and variations in children’s phonological WM performances. Hearing emotional stories, especially with negative contents, resulted in a significant attenuation of WM resources (Ellis & Ashbrook, 1988; Ellis & Moore, 1999; Spies et al., 1996). However, these results were also consistent with those of other studies (Gotoh, 2008; Greene & Noice, 1988; Kensinger & Corkin, 2003) suggesting that the effect of emotion on cognition is difficult to determine and to predict, particularly with children. For instance, in line with previous studies carried out with young adults (Levens & Phelps, 2008), positive emotion did not seem to affect children’s WM span.

The effect of emotion on cognition might be explained within the Cognitive Load Theory (Chanquoy, Tricot, & Sweller, 2007; Sweller, 1994), which distinguishes three types of load: intrinsic cognitive (referred to instructions), germane (concerned schemas), and extraneous cognitive load. The latter refers to the load generated by the way the information is presented to the participants. We believe that emotion could be considered as an extraneous cognitive load. Indeed, there are relatively consistent data indicating that heightened negative emotions have an impact on WM capacity, presumably by reducing cognitive resources (Ashcraft & Kirk, 2001; Fraser et al., 2012). For example, in a training task given to first-year medical students, Fraser and colleagues (2012) showed evidence that emotions could generate an increase in cognitive load and therefore a subsequent decline in performance.

Beyond the possibility of a cognitive load effect, other interpretations are possible. For instance, some studies showed that emotion could sometimes impair attentional resources whereas at other times it could enhance them (Anderson, 2005; Phelps, Ling, & Carrasco, 2006; Whalen, Bush, Shin, & Rauch, 2006). Other researchers reported evidence that happiness was task oriented whereas sadness was avoidance oriented. This means that emotion could be linked to a decline in motivation, which results in a decreased performance (Baas, De Dreu, & Nijstad, 2008; Carver & Harmon-Jones, 2009; Williams, Watts, MacLeod, & Mathews, 1997).

Finally, the effects of mood induction in children need to be confirmed by other studies. It would be interesting to assess the load emotion represents by administrating questionnaires in which participants could be asked to report the amount of mental effort invested during the task (Paas, Tuovinen, Tabbers, & Van Gerven, 2003). We also suggest investigating other valences such as fear, disgust or surprise, together with arousal and duration. Moreover, the mechanism responsible for these disturbances has yet to be identified. Although the hypothesis of intrusive thoughts can be advanced, as far as negative emotions are concerned, it still has to be proven. It could be simply an interfering inhibition state or a decrease in motivation. The results of the present study suggest that negative textual emotional content and the ensuing emotions generate cognitive overload, using up children’s cognitive resources.
